# The Development and Performance Validation of a Real-Time Stress Extraction Device for Deep Mining-Induced Stress

**DOI:** 10.3390/s26030875

**Published:** 2026-01-29

**Authors:** Bojia Xi, Pengfei Shan, Biao Jiao, Huicong Xu, Zheng Meng, Ke Yang, Zhongming Yan, Long Zhang

**Affiliations:** 1 College of Energy and Mining Engineering, Xi’an University of Science and Technology, Xi’an 710054, China; huicong.xu@xust.edu.cn (H.X.); 23203077044@stu.xust.edu.cn (Z.M.); 24203077046@stu.xust.edu.cn (K.Y.); 23203226083@stu.xust.edu.cn (L.Z.); 2 Yulin Institute of Green, Safe and Efficient Coal Mining and Clean Utilization, Xi’an University of Science and Technology, Yulin 719000, China; 3 Shaanxi Binchang Mining Group Co., Ltd., Xianyang 712000, China; 25203077040@stu.xust.edu.cn (B.J.); 22203226071@stu.xust.edu.cn (Z.Y.)

**Keywords:** deep mining, in situ stress monitoring, mining-induced stress evolution, laboratory experimental validation

## Abstract

**Highlights:**

**What are the main findings?**

- A novel in situ rock stress monitoring device was developed through structural optimization and material selection for deep and complex stress environments.
- Laboratory tests demonstrate a high consistency between monitored stress and applied stress, with peak error controlled within 5% and a fitting coefficient of R^2^ > 0.98.

**What are the implications of the main findings?**

- The proposed device provides a reliable method for real-time and accurate in situ stress monitoring in deep underground engineering.
- The findings offer technical support for stress evaluation and disaster prevention in deep mining and geotechnical engineering applications.

**Abstract:**

Under deep mining conditions, coal and rock masses are subjected to high in situ stress and strong mining-induced disturbances, leading to intensified stress unloading, concentration, and redistribution processes. The stability of surrounding rock is therefore closely related to mine safety. Direct, real-time, and continuous monitoring of in situ stress magnitude, orientation, and evolution is a critical requirement for deep underground engineering. To overcome the limitations of conventional stress monitoring methods under high-stress and strong-disturbance conditions, a novel in situ stress monitoring device was developed, and its performance was systematically verified through laboratory experiments. Typical unloading–reloading and biaxial unequal stress paths of deep surrounding rock were adopted. Tests were conducted on intact specimens and specimens with initial damage levels of 30%, 50%, and 70% to evaluate monitoring performance under different degradation conditions. The results show that the device can stably acquire strain signals throughout the entire loading–unloading process. The inverted monitoring stress exhibits high consistency with the loading system in terms of evolution trends and peak stress positions, with peak stress errors below 5% and correlation coefficients (R^2^) exceeding 0.95. Although more serious initial damage increases high-frequency fluctuations in the monitoring curves, the overall evolution pattern and unloading response remain stable. Combined acoustic emission results further confirm the reliability of the monitoring outcomes. These findings demonstrate that the proposed device enables accurate and dynamic in situ stress monitoring under deep mining conditions, providing a practical technical approach for surrounding rock stability analysis and disaster prevention.

## 1. Introduction

Coal resources in China are widely distributed and occur under complex geological conditions. Underground mining has long been the dominant extraction method, particularly in coalfields with burial depths exceeding 100 m [1,2,3,4,5]. With the continuous increase in mining depth and intensity, in situ stress levels in deep rock masses rise significantly, geological structures become increasingly complex, and the risks of surrounding rock deformation, failure, and dynamic disasters are markedly intensified, with stress evolution predominantly governed by mining-induced quasi-static redistribution while local dynamic disturbances may superimpose. Conventional ground pressure control and surrounding rock stability technologies developed for shallow or rigid mining conditions face severe challenges in deep, high-stress environments [6,7,8,9]. Consequently, safety issues associated with deep mining have become a critical factor restricting the efficient and sustainable development of coal mines.

In situ stress refers to the internal stress state that develops and remains stable within rock masses prior to excavation in coal mines and other underground engineering projects. It mainly originates from crustal tectonic movements, gravitational loading, and long-term geological evolution [10,11,12,13,14,15]. The in situ stress field determines the initial mechanical equilibrium of the surrounding rock and directly governs stress redistribution, deformation responses, and failure modes following excavation. Under deep mining conditions, increased burial depth, enhanced heterogeneity of the surrounding rock, and the expanded influence range of mining-induced disturbances cause the distribution and evolution of in situ stress to exhibit pronounced spatial variability and nonlinear characteristics. These features play a decisive role in roadway stability, support structure loading, and the initiation and evolution of mining-related disasters [16,17,18,19,20,21].

Accurately determining the magnitude, orientation, and evolution of in situ stress in coal and rock masses under deep mining conditions is therefore fundamental for elucidating surrounding rock deformation and failure mechanisms, optimizing mining and support parameters, and preventing dynamic disasters such as rock bursts. The geomechanical parameters of coal and rock masses mainly include in situ stress, rock mass strength, and structural parameters, which provide an essential basis for mine mechanical analysis, engineering design, and safety monitoring. In deep, high-stress, and strongly disturbed environments, surrounding rock stability depends not only on the magnitude and distribution of the initial stress field but also on stress release, redistribution, and localized structural damage during mining. Accordingly, the development of in situ stress monitoring techniques suitable for deep mining conditions is of considerable theoretical significance and engineering value [22,23,24,25,26].

Extensive studies worldwide have focused on the acquisition and evolution of in situ stress fields in coal mines and underground engineering, leading to the development of various monitoring methods and technical systems. In China, significant progress has been achieved in stress monitoring technologies and their engineering applications for complex stress environments in coal seams and surrounding rock. Techniques based on HI strain gauges have enabled quantitative characterization of stress release and redistribution in fractured zones, while microseismic monitoring has been widely applied to analyze mining-induced stress evolution through source localization and energy parameter inversion. In addition, in situ stress measurement methods using triaxial stress monitoring devices have been gradually developed, providing technical support for integrated measurements of stress magnitude and orientation. At the theoretical level, stress models incorporating microstructural characteristics have further improved the understanding of stress distribution under complex geological conditions [27,28,29,30].

Internationally, research has increasingly emphasized the coupling of rock mass failure mechanisms with multi-source monitoring technologies under deep, high in situ stress conditions. Borehole stress monitoring combined with fracture propagation analysis has provided new insights into mining-induced stress variations in coal seams. Meanwhile, integrated applications of microseismic monitoring and borehole imaging have been employed to investigate rock mass fracturing in large underground openings under high stress, revealing intrinsic relationships among fracture evolution, stress release, and failure modes from a multi-scale perspective [30,31,32,33]. These studies indicate that multiphysical data integration is an important direction for advancing the understanding of deep rock mass mechanical behavior.

Although substantial progress has been made in stress evolution analysis, monitoring techniques, and engineering applications, most existing approaches rely on indirect monitoring or staged measurements. Continuous, in situ acquisition of stress magnitude, orientation, and dynamic evolution during mining remains limited, particularly under high-stress, strongly disturbed conditions and in cases involving surrounding rock structural degradation [34,35,36]. The long-term stability and adaptability of stress monitoring devices under such conditions remain key challenges. Therefore, further development of in situ stress monitoring devices and methods suitable for deep mining environments is required to meet the demands for dynamic stress perception and safety control in complex underground engineering conditions.

## 2. Dynamic in Situ Stress Monitoring Device

To meet the requirements for in situ, long-term, and real-time monitoring of in situ stress in deep mining engineering, a novel in situ stress monitoring device suitable for borehole installation was developed in this study. The device is designed for high-risk areas, such as critical sections of deep roadways, stress-concentration zones, and regions surrounding goafs. It can be installed before or during mining operations to continuously monitor variations in surrounding rock stress, thereby providing fundamental data for surrounding rock stability analysis and engineering decision-making.

In practical engineering applications, the device is suitable for monitoring tasks under high-stress conditions, strong mining-induced disturbances, and complex borehole environments. After installation, the device can operate for extended periods during roadway excavation, mining advance, and goaf evolution, continuously recording stress variations in the surrounding rock. When the monitored region enters a stage of intense deformation or failure, the device may sustain partial damage. Nevertheless, the previously acquired continuous stress data remain effective for analyzing the characteristics of stress evolution. Therefore, the device can be deployed as a disposable or sacrificial monitoring unit in deep high-risk zones without compromising engineering safety or construction procedures.

During the strong deformation or failure stage of the surrounding rock, evident electrical signal anomalies, such as abrupt resistance variation in strain gauges, signal interruption, or persistent unstable noise, are used to identify functional damage of the monitoring device. Stress data acquired prior to the damage determination point are regarded as valid for stress evolution analysis and inversion, whereas data obtained after device failure are excluded. This criterion clearly defines the effective operating range of the device and facilitates reliable screening of valid monitoring data.

In addition, the device can be used independently for in situ stress monitoring or integrated into a multi-parameter monitoring system as a stress-sensing unit. It can be deployed in conjunction with microseismic, acoustic emission, or displacement monitoring techniques to identify abnormal stress variations and high-risk zones, thereby providing technical support for safety assessment and risk mitigation under deep mining conditions.

### 2.1. Principle of the Device

In this device, strain gauges are not directly bonded to the borehole wall, nor are they attached to a hollow capsule and subsequently fixed to the borehole wall with epoxy resin. Instead, a precise and reliable device is designed to transfer the borehole stress to the device’s interior. To ensure data accuracy, the transmitted stress should be as close as possible to the actual monitored stress. Therefore, the stress monitoring device must be capable of performing stress measurements directly within the borehole.

Stress is transmitted through a transmission medium to the strain gauges, which then convert it into directly observable strain data. Based on this principle, the following relationship can be derived:

The relationship between the resistance change (Δ*R*) of the strain gauge and the strain (*ε*) is given as:

(1) $$\varepsilon =\frac{\Delta R}{\begin{array}{l} R_{0}GF \end{array}}$$

where (*ε*) is the strain (dimensionless). (Δ*R*) is the change in electrical resistance of the strain gauge; (*R*_0_) is the initial resistance of the strain gauge. (*GF*) is the gauge factor of the strain gauge, which represents the change in resistance per unit strain.

For linear elastic materials, based on Hooke’s law, the relationship between stress (*σ*) and strain (*ε*) is expressed as:

(2) σ=Eε

where (*σ*) is the stress. (*E*) is the elastic modulus of the material. (*ε*) is the strain (dimensionless).

By combining the two equations, the relationship between stress and the resistance change in the strain gauge can be obtained as:

(3) $$\sigma =E\frac{\Delta R}{R_{0}GF}$$

This indicates that the strain in the material can be determined by measuring the change in the strain gauge’s resistance. The corresponding stress can then be calculated from the material’s elastic modulus.

### 2.2. Structure and Components of the Device

The core function of the monitoring device is to transfer in situ stress within the borehole through a transmission medium to locations on the device where strain can be effectively measured. The device is designed to ensure accurate stress transfer while protecting the strain gauges from external forces other than the intended stress transmission. Achieving precise stress transfer is therefore the primary challenge in the device design.

**Figure 1:**
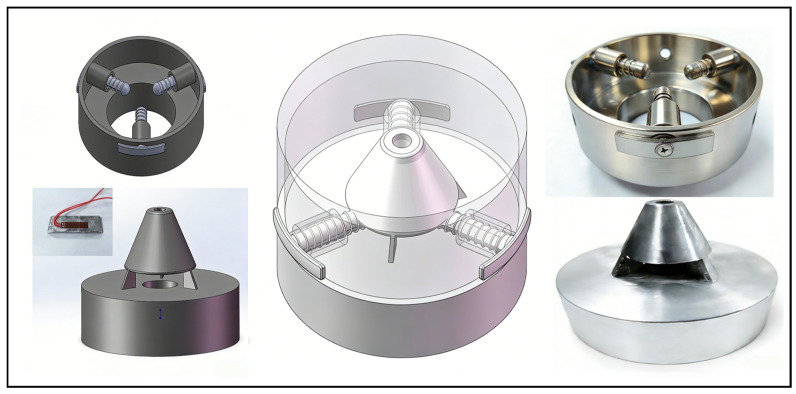
Structure of the in situ stress monitoring device.

As shown in Figure 1, the device comprises two main components: a probe and a base. The probe has a conical geometry that facilitates a reliable connection to other elements of the device. The circular disk connected beneath the probe serves as a gripping component during probe insertion before monitoring. After monitoring begins, it serves as a corrective measure. Three grooves are uniformly arranged around the probe’s conical head for installing strain blocks. After installing the strain blocks, the cross-section can be approximated as an equilateral triangle. Once assembled with the base, the probe–base system monitors in situ stress.

The base has a bowl-shaped geometry. After the borehole is drilled, the base is first moved to the designated monitoring position within the borehole. Once the base is installed correctly, the probe is subsequently inserted. After insertion into the base, a spring mounted on the positioning force transmission pin provides a preload, ensuring that the pin is oriented perpendicular to the strain block in space. The structural design of the positioning pin enables it to transfer the pressure from the borehole wall effectively. During operation, the base is first inserted and fixed inside the borehole. The probe is then inserted into the borehole via a push rod to achieve a tight coupling with the base.

The monitoring device is designed to be compatible with practical borehole conditions encountered in underground engineering. Its overall dimensions can be adjusted according to the borehole diameter, allowing the device to be customized for different drilling sizes without changing the core sensing structure. The installation relies on mechanical contact and structural confinement rather than precise geometric matching, which provides sufficient tolerance to variations in borehole diameter and minor deviations from ideal geometry. Consequently, the device can maintain stable stress transfer and monitoring performance in boreholes with rough walls, slight ovality, or local irregularities, demonstrating good adaptability to non-ideal borehole conditions commonly encountered in deep mining environments.

### 2.3. Design of the Strain Block

The strain block mainly consists of two components: a base material that meets the design requirements and strain-sensitive elements. The strain-sensitive elements are strain gauges of different models. The selection of strain gauges is based on their dimensions, electrical resistance, gauge factor, and applicable temperature range. The base material supports the strain-sensitive elements. This device is designed as an isosceles trapezoidal block with approximate dimensions of an upper base of 4 cm, a lower base of 5 cm, and a height of 1.2 cm.

Currently, commonly used strain gauges can be classified into five primary types: wire type, foil type, semiconductor, thin film, and thick film. Based on the substrate material and the apparent characteristics of strain blocks made of similar materials, the applicable operating conditions for different strain gauges were compared. In accordance with the design requirements, the BX120–20AA strain gauge was selected. Its main parameters are listed in Table 1.

The BX120-20AA foil strain gauge has a maximum allowable strain of 20,000 (*με*), which corresponds to a stress range exceeding the peak stress observed in the laboratory tests. Therefore, the strain gauge can capture stress evolution and concentration processes during deep mining before the occurrence of extreme failure.

In addition, the bonding layer firmly attaches the strain gauge to the base material surface, ensuring accurate strain transfer. To achieve uniform bonding between the strain gauge and the base material, a colorless, transparent adhesive with low viscosity and rapid curing characteristics should be selected. By comparing the bonding mechanisms and curing characteristics of several single- and two-component adhesives, including 502, 703, and 495, a combined adhesive system of 703 and 502 was selected. After curing, its elastic modulus lies between that of the strain-gauge substrate and that of a similar material, enabling accurate transfer of the strain block’s deformation. The combined use of 502 adhesive and 703 silicone rubber provides both high initial bonding strength and long-term moisture resistance, ensuring stable strain transfer under simulated underground humid conditions.

Strain gauge bonding was performed as follows. First, the bonding surface was cleaned with alcohol to remove oil, grease, and contaminants. Positioning lines were marked on the strain block at the designated bonding location. The strain gauge was positioned and temporarily fixed using transparent adhesive tape, with the tape extending slightly beyond the gauge length. After alignment, the strain gauge was lifted, a drop of 502 adhesive was applied, and the indicator was repositioned. A transparent plastic film was placed over the gauge, and uniform pressure was applied for approximately one minute. The plastic film was removed, and the strain-gauge surface was cleaned with alcohol, thereby completing the bonding process. The electrical resistance between the two lead wires was measured to verify that it matched the wire resistance, thereby ensuring secure soldering and the absence of short circuits. When the lead wire length is within 2 m, the wire resistance can be neglected. For long-term use, the bonded strain gauge was protected by applying transparent 705 adhesives over the gauge surface, which cures within 24 h.

Moisture protection for the strain block is essential, as stress monitoring devices are often exposed to high-temperature, high-humidity environments. After comparing various commercial adhesives, 703 room-temperature vulcanized (RTV) silicone rubber was selected. This material is solvent-free and non-corrosive, with a surface curing time ranging from 3 to 30 min, depending on temperature. It exhibits strong adhesion, electrical insulation, moisture resistance, vibration resistance, aging resistance, arc resistance, and oil resistance, and can operate normally over a temperature range from −60 °C to +250 °C. During strain block fabrication, after the strain gauges were bonded, the silicone rubber was uniformly applied over the strain gauges and exposed solder joints. The assembly was then cured at room temperature for 24 h before use.

### 2.4. Finite Element Analysis of the Mechanical Response and Failure Evolution of the Monitoring Device

Finite element analysis (FEA) was employed as a numerical simulation and virtual prototyping tool to evaluate the structural rationality of the monitoring device. In FEA, complex geometries are discretized into a finite number of elements, and the mechanical response of the structure under external loading is simulated based on governing equations. This approach is widely used for strength verification and stiffness assessment, enabling design reliability to be evaluated beyond physical prototypes.

In this study, a three-dimensional solid finite element model was established to analyze the stress and deformation characteristics of the monitoring device under confining pressure. All components were described using linear elastic constitutive models, with material properties defined by the elastic modulus and Poisson’s ratio of the corresponding metallic materials. Plastic deformation and damage evolution were not considered, as the analysis focused on the elastic response of the device within its designed working load range.

**Figure 2:**
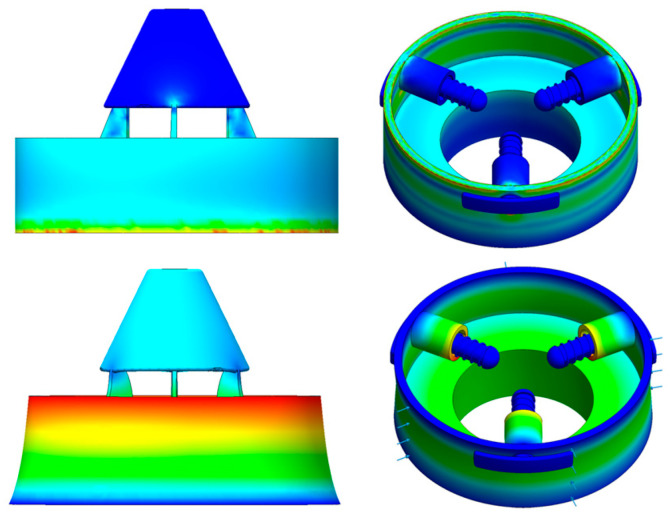
Compressive stress contour of the monitoring device.

As shown in Figure 2, the finite element deformation contour of the monitoring device under confining pressure and the corresponding comparison results are presented. The results indicate that the device undergoes a certain degree of elastic deformation under external confining pressure. The deformation is primarily concentrated in the outer casing, which is in contact with the surrounding medium, and in regions of local stress transition. In contrast, the device’s internal key functional units remain structurally stable.

Comparison of the results before and after confining pressure loading shows a clear overall deformation trend of the device. However, the deformation distribution remains continuous, with no local discontinuities or pronounced stress concentrations. This indicates that the device operates within the elastic regime under the applied loading conditions. The outer casing transmits the external confining pressure through global load-bearing, enabling smooth transfer of the load within the structure. This mechanism effectively prevents overload-induced failure in locally weak regions.

Further analysis indicates that the device’s deformation is primarily characterized by overall contraction and local displacement variations. No structural instability or relative misalignment of functional components is observed. Provided that no structural damage occurs, this type of deformation can be regarded as controlled elastic deformation. It does not adversely affect the internal stress-transfer path or the device’s strain-acquisition mechanism. Conversely, a moderate degree of global deformation facilitates uniform transmission of external confining pressure into the device. This enables the strain-sensitive elements to respond stably to variations in external stress.

In addition, the contour distributions indicate that stress and deformation are relatively uniform throughout the device. This demonstrates that the structural design exhibits good global stiffness and load-bearing capacity under confining pressure conditions. These results suggest that, under confining pressure in the surrounding deep rock, the monitoring device can maintain its core monitoring functionality even when a certain degree of structural deformation occurs. Local deformation does not compromise the effectiveness or reliability of in situ stress monitoring.

In summary, the finite element analysis results confirm that the monitoring device maintains good structural integrity and mechanical stability under confining pressure. The induced elastic deformation does not impair its stress monitoring capability. These findings provide a reliable mechanical basis for the engineering application of the device in deep and complex stress environments.

**Figure 3:**
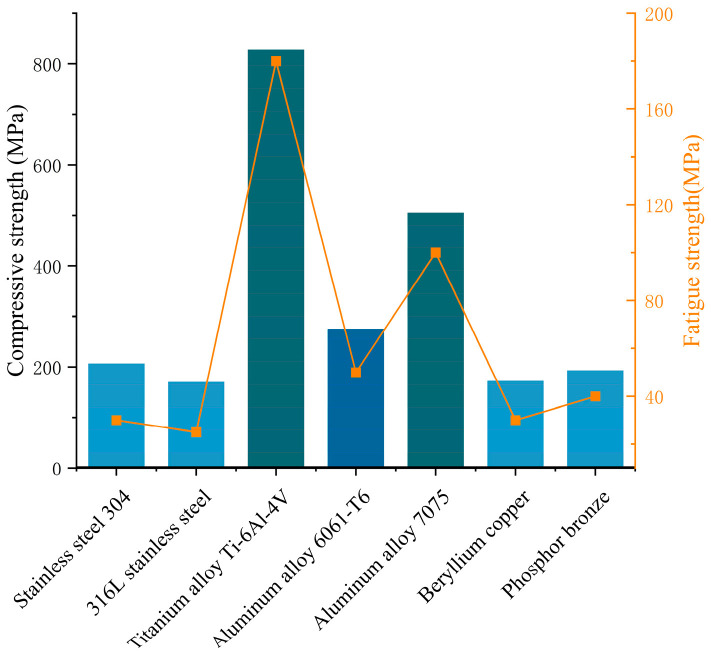
Comparison of strain block materials.

To optimize the device’s structural performance and improve its long-term stability, a comparative analysis of commonly used metallic materials was conducted. Attention was paid to differences in the compressive and fatigue strengths of various metals when applied to the strain block. As shown in Figure 3, when a point load is used at the center of the strain block, the titanium alloy exhibits the highest yield strength, reaching 827 MPa, indicating excellent load-bearing capacity. Aluminum alloy follows, with a yield strength of approximately 275 MPa. The yield strengths of materials such as stainless steel and beryllium copper are relatively lower, generally around 200 MPa. Further comparison of fatigue strength indicates a positive correlation with compressive strength among the investigated metals. Titanium alloy again exhibits the most outstanding fatigue performance, followed by aluminum alloy; the remaining materials exhibit relatively low fatigue resistance. These results indicate that titanium alloy offers significant advantages in ensuring both the device’s strength and durability, making it the preferred material for strain block fabrication.

**Figure 4:**
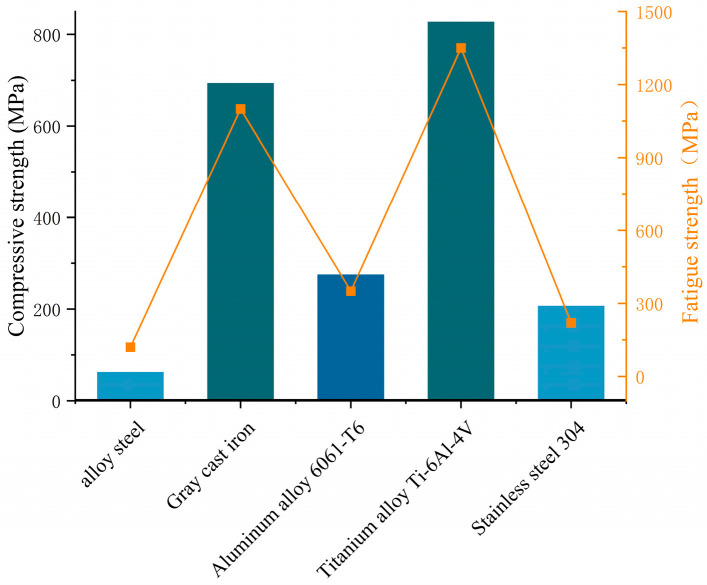
Comparison of materials for the base and probe.

The components of the device, excluding the strain block, can be regarded as the casing, which provides mechanical support and protection for the strain block. The casing material was selected through a comparative analysis of commonly used metallic materials. As shown in Figure 4, gray cast iron exhibits a compressive strength of 693 MPa, whereas the titanium alloy reaches 827 MPa, both demonstrating relatively high strength. Aluminum alloy and stainless steel show moderate compressive strength, whereas alloy steel exhibits the lowest strength. The fatigue strength of these materials also shows a proportional relationship with compressive strength.

Considering strength, corrosion resistance, and economic efficiency, the titanium alloy was ultimately selected as the material for the strain block. Gray cast iron was selected as the material for the base and the probe. In terms of structural design, the device adopts an integrated metallic encapsulation. A stable cylindrical monitoring unit is formed inside the borehole through the modular assembly of the probe and the base, which effectively enhances adaptability during borehole installation and resistance to mechanical damage. The strain-sensitive elements are indirectly coupled with external rock mass deformation through force transmission components, preventing direct exposure of the strain gauges to the borehole wall environment. This structural design reduces the adverse effects of point load impacts, borehole wall damage, and water intrusion on the sensing elements, thereby ensuring long-term reliable monitoring.

In terms of material selection, the load-bearing capacity, durability, and deformation compatibility under deep mining conditions were comprehensively considered. Based on these factors, targeted materials were selected for the key components of the device. By appropriately matching the mechanical properties of structural materials and force transmission components, rock mass stress can be effectively transferred and converted into identifiable strain signals. From a material perspective, this approach enhances the measurement sensitivity and stability of the device.

On this basis, finite element numerical analysis was employed to systematically investigate the stress state and deformation characteristics of the device under different loading conditions. The results show that under typical stress conditions, the device exhibits rational load-bearing behavior and a uniform deformation distribution. The strain concentration regions are well matched with the locations of the sensing elements, confirming the rationality and feasibility of the structural design and material selection. The finite element analysis results provide a reliable basis for the final determination of structural parameters and subsequent experimental validation of the device.

In summary, this chapter demonstrates the design of the in situ stress monitoring device from three aspects: structural configuration, material performance, and numerical simulation. These analyses establish a solid foundation for subsequent laboratory validation and performance evaluation.

## 3. Laboratory Validation

Under deep mining conditions, the stress state of coal and rock masses ahead of roadways or mining faces undergoes significant disturbance and redistribution. As roadway excavation or mining advance proceeds, the initially stable in situ stress field is disturbed by excavation activities. The stress in the surrounding rock gradually evolves from an initial equilibrium state to a non-uniform distribution. Compared with shallow mining, coal and rock masses under deep mining conditions are more sensitive to stress disturbances due to the high in situ stress background. Their stress paths typically exhibit pronounced staged characteristics.

Under excavation-induced disturbance, the radial (horizontal) stress of the surrounding rock in the near-roadway zone decreases rapidly. In some local areas, the radial stress approaches zero, whereas the axial stress increases significantly due to stress concentration effects. When the axial stress exceeds the load-bearing capacity of the coal–rock structure, crack propagation and structural failure occur within the surrounding rock. This process manifests as typical deformation and failure phenomena, such as rib spalling, floor heave, and expansion of the loosening zone. The failure process is accompanied by rapid local stress unloading, causing the surrounding rock to enter a new stage of stress redistribution and structural reorganization after failure.

In regions far from the working face, the influence of excavation-induced disturbance gradually attenuates. The stress state of the surrounding rock remains dominated by the original in situ stress field, with relatively small variations in radial and axial stresses, and the overall stability remains high. Consequently, under deep mining conditions, the stress distribution of the surrounding rock typically exhibits a spatial zoning pattern characterized by “near-field unloading, stress concentration ahead of the face, and relatively stable far-field conditions.” This stress evolution pattern has been validated by numerous numerical simulations, physical model experiments, and field observations.

Based on the typical stress evolution characteristics under deep mining conditions described above, the validation tests of the stress monitoring device simulated a loading–unloading path in which the surrounding rock transitions from an initial uniform stress state to a biaxial unequal stress condition with high axial stress concentration. This design ensures that the specimen’s stress state during testing closely approximates the actual loading conditions experienced by the surrounding deep rock during excavation. After completion of the tests, the data recorded by the monitoring device were collected. The corresponding stress values were then inverted using the stress–strain relationship and compared with the actual loading data recorded by the loading system and the acoustic emission monitoring results. This comparative analysis was used to comprehensively evaluate the reliability, sensitivity, and engineering applicability of the in situ stress monitoring device.

By conducting validation tests using typical stress paths representative of actual deep mining conditions, the working performance of the stress monitoring device under high stress, strong disturbance, and unloading conditions can be more realistically evaluated. This approach ensures that the experimental conclusions have strong engineering relevance and practical applicability.

**Figure 5:**
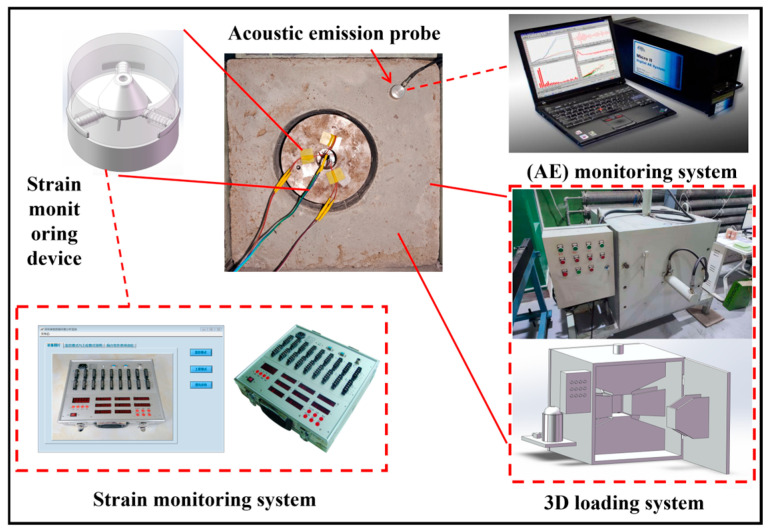
Schematic diagram of the experimental system.

After the tests were completed, strain data were collected and converted into applied stress values using the corresponding calculation formulas. The inverted stress values were then compared with the data obtained from the loading system and the acoustic emission monitoring system. These results were used to evaluate the monitoring accuracy and practicality of the stress monitoring device under complex stress conditions, thereby verifying its feasibility for application in simulated roadway surrounding rock stress environments. The data acquisition system operates in a continuous sampling mode to achieve real-time, second-level monitoring of stress evolution in the surrounding rock. The selected sampling frequency is sufficient to continuously capture the key stages of stress evolution during deep mining, including stress loading, concentration, unloading, and redistribution. It should be noted that the term “real-time” in this study refers to the continuous and synchronous acquisition of stress evolution processes, rather than millisecond-level responses to instantaneous microcrack propagation. The monitoring objective, therefore, focuses on the macroscopic evolution and concentration characteristics of mining-induced stress. The experimental system is shown in Figure 5.

### 3.1. Three-Dimensional Stress Loading System

The main technical parameters of the three-dimensional stress loading system are listed in Table 2. The system comprises a cubic frame, a stress-loading system, a hydraulic servo system, a data acquisition system, and a control system. The experimental system is capable of simultaneously applying pressures ranging from 0 to 8 MPa along the X, Y, and Z directions of the specimen.

The entire stress loading system is constructed using a cubic frame structure. As shown in the figure, a pair of oppositely arranged hydraulic loading units is installed between the base and the top plate along the vertical direction to apply and precisely control axial stress. Another pair of oppositely arranged hydraulic loading units is installed on the left and right sides of the frame to apply horizontal confining pressure. Openable doors are arranged at the front and rear of the system to serve as access passages for specimen installation and removal. After closure, the doors are secured by four sets of high-strength bolts to ensure structural stability and monitoring accuracy during loading in this direction.

The hydraulic servo system is the core power module of the apparatus. Its primary function is to achieve automatic, rapid, and precise control of output variables, such as force, displacement, and velocity. The system employs servo valves to perform distributed control of three hydraulic pump units, enabling independent pressurization and depressurization along each loading direction. When a loading command is issued by the control system, the hydraulic pumps rotate forward and pressurize the hydraulic oil in the pipelines. The pressure is transmitted through pressure lines to the stress loading modules, thereby applying load to the specimen. When the hydraulic pumps rotate in reverse, the pressure is gradually released, realizing the unloading process.

The data acquisition system consists of sensors, signal acquisition units, and a computer terminal. Sensors are installed on the hydraulic cylinder pressure rods and within the hydraulic pump system to monitor stress and displacement variations in real time during loading and unloading processes. The acquired signals are transmitted to the computer system through the data acquisition units and converted into pressure values along different directions. These data characterize the stress evolution of the specimen throughout the entire loading process and provide fundamental data for the performance evaluation of the stress monitoring device.

The control system is responsible for coordinating the loading and unloading processes of the hydraulic servo system. The system controls the operating direction of the hydraulic pumps through electrical circuits, while human–machine interaction is achieved via multi-directional control buttons on the control panel. During the experiment, operators can precisely regulate electrical signals through button commands to accomplish pressurization, pressure holding, and depressurization. This ensures accurate and controllable operation of the stress loading system throughout the entire testing process.

### 3.2. Experimental Program

The specimens used in the experiments were prepared by casting concrete material. Cement, sand, and other raw materials were mixed thoroughly according to the designed proportions and then poured into molds. A release agent was uniformly applied to the inner surfaces of the molds in advance. The specimen dimensions were 300 mm × 300 mm × 300 mm. A through-hole with a diameter of 114 mm was prefabricated at the center to accommodate the in situ stress monitoring device. After casting and initial setting, the specimens were demolded and cured in a constant-temperature and constant-humidity chamber for 30 days to ensure stable mechanical properties. The specimen size was selected to ensure sufficient boundary distance relative to the borehole diameter, thereby minimizing boundary effects on the stress transfer and fracture response.

Cement-based specimens were adopted in this study mainly for experimental controllability and repeatability. Compared with natural coal or rock masses, cement-based materials allow systematic control of initial damage levels, loading paths, and boundary conditions under laboratory conditions, thereby focusing the validation on the performance of the stress monitoring device. It should be noted that the objective of this study is to evaluate the monitoring reliability under complex stress and damage conditions, rather than to directly characterize the mechanical properties of natural rock masses.

Before the formal tests, Specimen No. 1 was selected for a conventional compression failure test. The specimen was loaded to complete failure without any initial damage to determine its maximum compressive strength. The results show that Specimen No. 1 experienced overall failure when the applied stress reached 5.31 MPa. Based on this reference value, the remaining three specimens were subjected to different initial damage preconditioning levels, corresponding to 30%, 50%, and 70% of the maximum compressive strength. The corresponding loading stresses were 1.59 MPa, 2.65 MPa, and 3.71 MPa, yielding three specimens with varying degrees of initial damage. After preconditioning, all specimens remained structurally intact and were used for subsequent loading tests.

During the formal tests, the assembled in situ stress monitoring device was inserted into the prefabricated through-hole of the specimen, and the specimen was then placed in the three-dimensional stress-loading system. The position of the specimen was adjusted to ensure that the axis of the through-hole was aligned with the *Z*-axis of the loading system. The lead wires of the strain gauges were routed through the reserved observation port on the end cover of the loading system and connected to a static resistance strain meter.

Acoustic emission (AE) monitoring was carried out using a single AE sensor. The sensor was installed on the specimen surface on the side containing the borehole and was coupled to the specimen using hot-melt adhesive to ensure stable signal transmission during loading. Throughout the experiment, the AE system continuously recorded acoustic emission activity synchronously with stress loading, providing information on crack initiation and damage evolution in the coal–rock specimen. The recorded AE energy and ringing count were subsequently used to analyze the correspondence between microscopic damage development and macroscopic stress evolution.

Prior to the start of the experiment, the front and rear end covers of the loading system were closed and secured. The power supply was then switched on, and the three-directional hydraulic cylinders were activated to check the operating status of the three-dimensional stress loading system. Subsequently, the static resistance strain meter and the acoustic emission monitoring system were activated. After confirming proper channel connections and stable signal acquisition, the loading stage was initiated.

During loading, the three-dimensional stress system applied a confining pressure to all specimens at a constant loading rate until complete failure, thereby ensuring comparability of experimental results across different initial damage conditions. Throughout the tests, the acoustic emission monitoring system recorded variations in acoustic emission energy and ringing counts in real time. Meanwhile, the stress monitoring device synchronously acquired strain signals, reflecting the stress state and deformation response of the specimens during loading. After completion of each test, the monitoring data were checked and processed. Once data validity was confirmed, the stress monitoring device and acoustic emission sensors were removed, and the same procedure was repeated for the remaining specimens.

## 4. Experimental Results

### 4.1. Fracture Results of Specimens with Different Initial Damage Levels

At the initial stage under confining pressure, the cement specimens remain relatively stable. Acoustic emission monitoring indicates that internal energy fluctuations are small and insufficient to induce structural damage. The stress monitoring device records a gradual increase in stress magnitude, accompanied by an increasing stress growth rate.

When the confining pressure approaches approximately 3 MPa, microcracks begin to initiate within the specimen. At this stage, the acoustic emission monitoring system records a pronounced increase in energy fluctuations. Correspondingly, the stress monitoring device shows a rapid rise in the monitored stress curve. With further increases in confining pressure, additional minor cracks gradually develop. When the confining pressure reaches approximately 5 MPa, the specimen does not fail. Instead, a dominant crack forms parallel to the cross-sectional plane of the cylindrical surface of the central borehole, accompanied by several induced microcracks in its vicinity.

**Figure 6:**
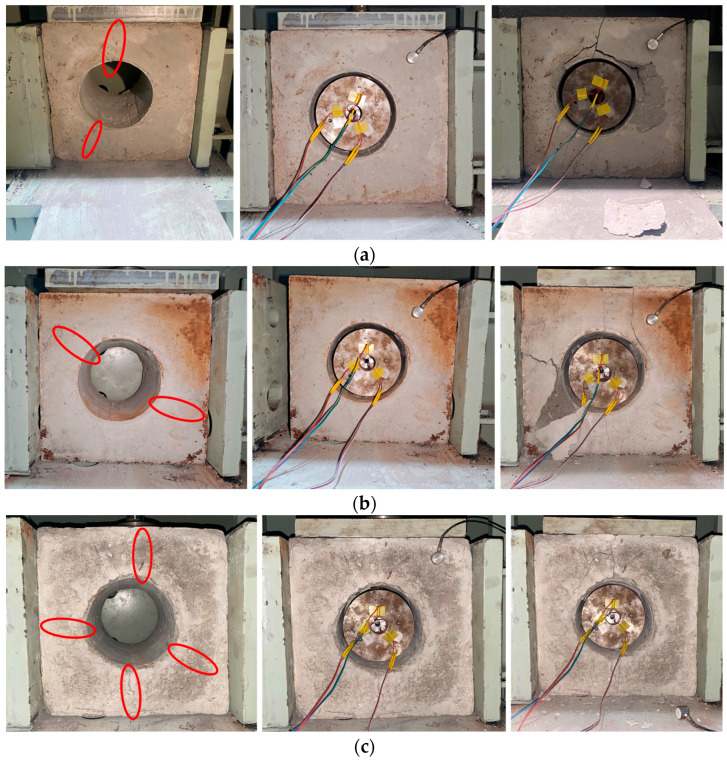
Comparison of specimens before and after fracturing: (**a**) Sample No. 2; (**b**) Sample No. 3; (**c**) Sample No. 4.

When the confining pressure increases to approximately 5 MPa, splitting cracks develop in the specimen, with fracture planes parallel to the cross-sectional plane of the side surface of the central borehole. A large number of internal cracks form, and the specimen’s structural integrity is destroyed, resulting in irreversible damage. Specimens No. 2, No. 3, and No. 4 were subjected to initial damage preconditioning in advance, and cracks of varying degrees were observed on these specimens. During loading, the monitored data for these specimens show notable differences relative to those of Specimen No. 1, which had no initial damage and was made of the same material. These three specimens exhibit relatively high acoustic emission energy levels from the initial loading stage and enter the rapid energy growth phase earlier than Specimen No. 1 under continuous loading. However, due to the presence of pre-existing damage, their energy peak values are slightly lower than those of Specimen No. 1, and the energy release is more concentrated. The energy peak of Specimen No. 2 is lower than that of Specimen No. 1 and occurs at an earlier loading stage. For Specimen No. 4, with an initial damage level of 70%, no pronounced high-energy release peak is observed. Instead, multiple smaller energy release peaks occur. Figure 6 shows samples 2, 3, and 4, with the cracks indicated in the figure.

#### 4.1.1. Acoustic Emission Analysis of Specimens with Different Initial Damage Levels

**Figure 7:**
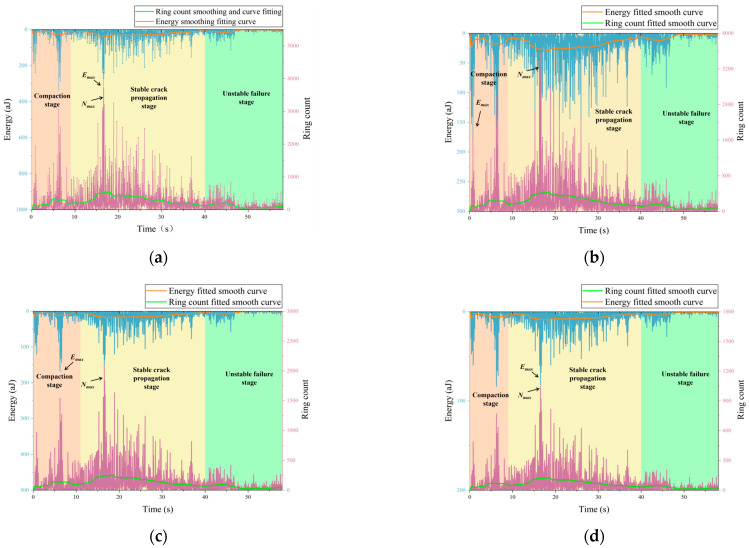
Acoustic emission monitoring results of the specimens: (**a**) Specimen without initial damage; (**b**) Specimen with 30% initial damage; (**c**) Specimen with 50% initial damage; (**d**) Specimen with 70% initial damage.

Figure 7 presents the time-dependent evolution of acoustic emission (AE) energy and ringing counts for specimens with no initial damage and with initial damage levels of 30%, 50%, and 70% during the fracturing loading process. By combining the temporal variations in AE energy and ringing counts with their smoothed fitting curves, the AE response of each specimen can be divided into three stages: a compaction stage, a stable crack propagation stage, and an unstable failure stage.

As shown in Figure 7a, the specimen without initial damage exhibits weak AE activity during the early loading stage. Both AE energy and ringing counts remain at low levels, with only sporadic fluctuations observed near the end of the compaction stage, indicating that this stage is dominated by the closure of pre-existing microcracks. Upon entering the stable crack propagation stage, AE events gradually increase. AE energy and ringing counts begin to show intermittent peak behavior, while the overall distribution remains relatively concentrated. With further loading, during the unstable failure stage, both AE energy and ringing counts exhibit pronounced burst-like increases, characterized by dense peaks with large amplitudes.

As shown in Figure 7b, compared with the undamaged specimen, the specimen with 30% initial damage exhibits a significantly earlier onset of AE activity. Frequent ringing counts and energy fluctuations are observed even during the compaction stage, indicating that pre-existing damaged cracks are activated at an early loading stage. During the stable crack propagation stage, the number of AE events increases markedly, and the occurrence frequency of energy peaks rises. However, the amplitude of individual peaks is lower than that of the undamaged specimen. In the unstable failure stage, relatively concentrated energy release can still be observed, but the burst intensity is reduced. This indicates that slight initial damage weakens the energy concentration capacity, causing the failure mode to shift from strongly abrupt to relatively moderate.

As shown in Figure 7c, when the initial damage level increases to 50%, the AE activity exhibits a more pronounced full-process characteristic. AE events occur throughout nearly the entire loading process, and the boundary between the compaction stage and the stable crack propagation stage becomes indistinct. The peak distributions of AE energy and ringing counts become more dispersed. The energy of individual events is significantly lower than that of specimens with lower damage levels, while the event occurrence frequency further increases. During the unstable failure stage, no pronounced concentrated burst is observed. Instead, sustained releases of multiple moderate-energy events occur, indicating that the internal crack network has developed a certain degree of connectivity and that the failure process is dominated by progressive crack growth.

As shown in Figure 7d, the specimen with 70% initial damage exhibits the most pronounced differences in AE behavior. AE activity is highly active from the early loading stage, with frequent ringing counts and energy signals, but the overall amplitudes remain low. With continued loading, neither AE energy nor ringing counts show a distinct concentrated growth stage, and the characteristics of the unstable failure stage are no longer evident. The AE response is characterized by a “high-frequency–low-energy–continuous release” pattern. This behavior indicates that under high initial damage conditions, the structural integrity of the coal–rock mass is severely degraded. The failure process is mainly governed by crack sliding and local crushing, making it difficult to form large-scale energy accumulation and instantaneous release.

A comprehensive comparison of the AE responses of the four specimen groups shows that, with increasing initial damage, the onset time of AE activity occurs progressively earlier, and the AE behavior gradually transitions from concentrated bursts prior to failure to continuous release throughout the entire loading process. Meanwhile, the peak values of AE energy and ringing counts for individual events continuously decrease, whereas the event occurrence frequency increases markedly. Overall, the AE characteristics exhibit an evolutionary pattern characterized by an earlier onset, enhanced activity over the loading process, dispersed peak values, and weakened instability-related bursts.

#### 4.1.2. Stress Monitoring Analysis of Specimens with Different Initial Damage Levels

The monitoring device contains three strain blocks that are uniformly distributed along the circumferential direction. Their spatial arrangement can be approximated as an equilateral triangular configuration located in the same plane, with an angular spacing of 120° between adjacent blocks. Under external confining pressure or axial loading, the applied loads are transmitted to the three strain blocks through the structural load-transfer paths.

Owing to differences in installation positions and load-transfer directions, the magnitudes and directions of the forces acting on each strain block are not identical.

**Figure 8:**
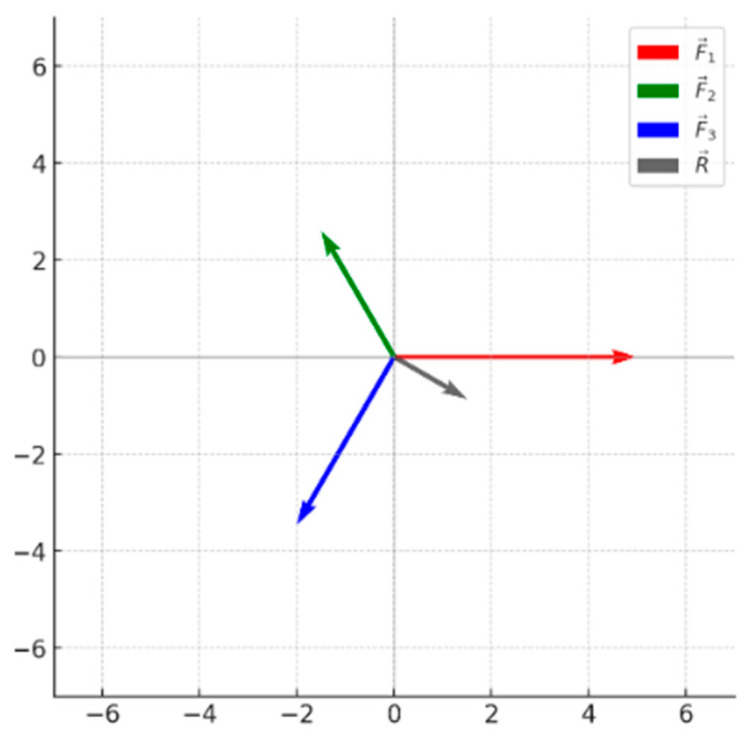
Schematic diagram of stress distribution.

These forces are denoted as, respectively, as ($\vec{F_{1}}$), ($\vec{F_{2}}$), and ($\vec{F_{3}}$) illustrated in Figure 8.

From a physical perspective, these three forces act along the three sides of the equilateral triangular configuration within the device, reflecting the decomposition and transmission characteristics of external stress inside the structure. To facilitate the establishment of a unified mechanical analysis model and to highlight the overall loading characteristics of the device, an equivalent mechanical approach is adopted. The three distributed forces are transformed into an equivalent concentrated force system acting at a common reference point.

In rigid body and structural mechanics, when the object of interest is global deformation and stress response, and the dominant effect of the external force system is overall deformation rather than local rotation or bending, forces acting at different locations but within the same plane can be equivalently translated to a single point without altering their mechanical effects. In the present study, the monitoring device undergoes predominantly global elastic deformation under confining pressure. The forces acting on the three strain blocks jointly contribute to the overall loading and deformation of the device through structural constraints, while local moment effects have a negligible influence on the monitoring function. Therefore, it is reasonable to translate and concentrate ($\vec{F_{1}}$), ($\vec{F_{2}}$), ($\vec{F_{3}}$) to a common point for resultant force analysis.

(4) $$\vec{F_{1}}=\left(F_{1},0\right)$$

(5) $$\vec{F_{2}}=\left(F_{2}\cos120{}^{\circ},F_{2}\sin120{}^{\circ}\right)=\left(-\frac{1}{2}F_{2},\frac{\sqrt{3}}{2}F_{2}\right)$$

(6) $$\vec{F_{3}}=\left(F_{3}\cos240{}^{\circ},F_{3}\sin240{}^{\circ}\right)=\left(-\frac{1}{2}F_{3},-\frac{\sqrt{3}}{2}F_{3}\right)$$

On this basis, the equivalent force acting on the device can be expressed as the vector superposition of the three forces, i.e.,

(7) $$R_{x}=F_{1}-\frac{1}{2}\left(F_{2}+F_{3}\right)$$

(8) $$R_{y}=\frac{\sqrt{3}}{2}\left(F_{2}-F_{3}\right)$$

The resultant force vector is given by:

(9) $$\vec{R}=\left(R_{x},R_{y}\right)$$

The magnitude of the resultant force is given by:

(10) $$\vec{\left|R\right|}=\sqrt{R_{x}^{2}+R_{y}^{2}}=\sqrt{{\left(F_{1}-\frac{F_{2}+F_{3}}{2}\right)}^{2}+\frac{3}{4}{\left(F_{2}-F_{3}\right)}^{2}}$$

Here, $\vec{R}$ denotes the equivalent resultant force acting on the strain block system inside the device under external loading. This resultant force characterizes the combined mechanical effects induced by external confining pressure and axial loading within the device, and serves as a key basis for analyzing the overall stress state and strain response characteristics of the monitoring system.

The stress inversion method adopted in this study is based on linear elasticity and equivalent force superposition. Its applicability assumes that the monitoring device operates within the elastic regime, that internal bending or relative sliding is negligible, and that external stress is transferred to the strain blocks in an approximately uniform manner. Within the loading range considered, finite element analysis indicates that the device undergoes overall elastic deformation without pronounced local buckling or contact nonlinearity.

For specimens with initial damage levels of 50–70%, crack development may introduce local contact variations and non-uniform stress transfer, which mainly manifest as enhanced high-frequency fluctuations in the monitored stress signals and constitute a source of uncertainty in stress inversion. However, prior to functional damage of the device, these uncertainties primarily affect instantaneous fluctuations rather than the overall stress evolution trend or peak response. Therefore, before the damage determination point, the linear elastic-based inversion method remains applicable at the macroscopic scale.

**Figure 9:**
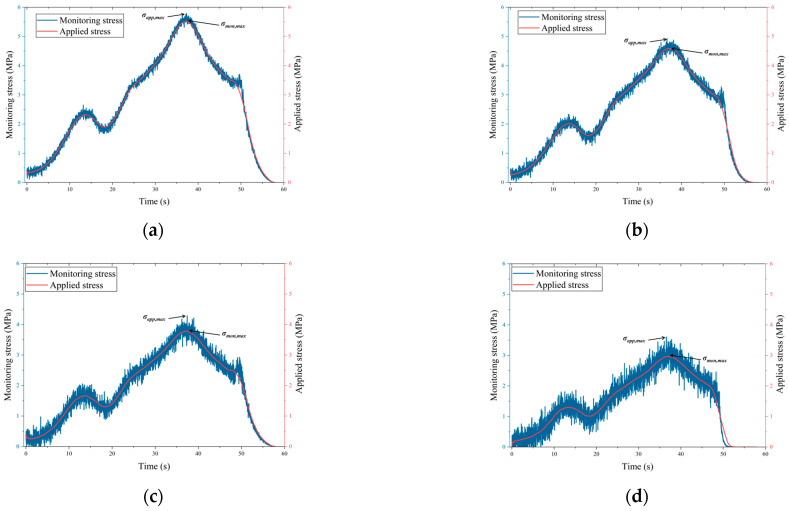
Comparison of stress monitoring results of the test samples: (**a**) Specimen without initial damage; (**b**) Specimen with 30% initial damage; (**c**) Specimen with 50% initial damage; (**d**) Specimen with 70% initial damage.

Figure 9 presents the comparison between applied stress and monitored stress for specimens with no initial damage and with initial damage levels of 30%, 50%, and 70% during the loading–unloading process. In the figure, the red curves represent the applied stress recorded by the loading system, while the blue curves denote the monitored stress inverted from the stress monitoring device.

As shown in Figure 9a, under the condition of no initial damage, the monitored stress remains highly consistent with the applied stress throughout the entire loading–unloading process. During the loading stage, the two curves exhibit good synchrony in terms of stress increase, local fluctuations, and peak stress, with only minor high-frequency fluctuations observed locally. These fluctuations mainly result from transient stress adjustments caused by the initiation and closure of microcracks within the specimen, while the overall trend remains consistent with the applied stress. During the unloading stage, the monitored stress decreases rapidly and continuously in response to the applied stress, without noticeable lag or distortion.

As shown in Figure 9b, when the specimen has an initial damage level of 30%, the overall evolution trend of the monitored stress remains in good agreement with that of the applied stress. Compared with the undamaged specimen, the amplitude of high-frequency fluctuations in the monitored stress curve slightly increases, particularly during the middle and late stages of loading. This behavior indicates that pre-existing cracks are activated under external loading, leading to more frequent local stress redistribution. However, in terms of peak stress location, peak magnitude, and unloading behavior, the monitored stress can still accurately capture the variations in the applied stress, without systematic deviation. This demonstrates that the device maintains good monitoring performance under mild damage conditions.

As shown in Figure 9c, under the condition of 50% initial damage, the fluctuation characteristics of the monitored stress curve are further intensified. During the middle and late stages of loading, the dispersion of the blue curve is markedly greater than that observed in specimens with lower damage levels. This behavior indicates that, with increasing crack connectivity, the internal stress transfer paths become more complex, and local damage and sliding events occur more frequently. Nevertheless, in terms of the overall trend, the monitored stress can still effectively track the increase, peak, and decrease in the applied stress. The two curves are generally consistent in both peak stress magnitude and peak occurrence time. During the unloading stage, the monitored stress also responds rapidly, indicating that the device maintains good adaptability under moderate damage conditions.

As shown in Figure 9d, under the condition of 70% initial damage, the monitored stress curve exhibits the most pronounced fluctuation characteristics. During loading, the amplitude of high-frequency fluctuations in the monitored stress increases significantly, indicating that the internal structure of the specimen is highly fractured and that stress transfer is dominated by repeated local adjustments and sliding events. Despite this, the monitored stress remains consistent with the applied stress in terms of the macroscopic trend and can clearly capture the stages of stress increase, peak, and unloading. During the rapid unloading stage, no obvious lag is observed in the monitored stress, indicating that the device can operate stably under high damage and strong disturbance conditions, and that its core measurement function does not fail even as specimen damage increases.

A comparative analysis under different initial damage conditions indicates that, with increasing initial damage, the high-frequency fluctuations of the monitored stress curves become progressively more pronounced, reflecting enhanced signal disturbance caused by crack development and local damage within the coal–rock mass. These high-frequency fluctuations are mainly attributed to stress adjustments induced by crack initiation, propagation, and local slip, rather than internal mechanical slippage or instability of the monitoring device. On the one hand, the device adopts an integrated metal-encapsulated structure, and finite element analysis confirms that it maintains good structural integrity during loading. On the other hand, the stress fluctuations show good temporal correspondence with the evolution of acoustic emission energy and ringing counts, indicating that the observed fluctuations primarily reflect rock mass fracture activity.

Despite the increased signal fluctuations, the monitored stress remains in good agreement with the applied stress under all initial damage conditions in terms of overall evolution trend, peak stress occurrence, and unloading response. The relative error of the peak stress is controlled within 5%, and the fitting correlation coefficients (R^2^) between the monitored and applied stresses are all greater than 0.95, with no systematic deviation or obvious lag observed. These results demonstrate that the developed in situ stress monitoring device can achieve high-accuracy stress measurement under intact structural conditions and maintain good stability and reliability under varying degrees of initial damage and complex internal disturbance conditions.

The coupling between acoustic emission activity and stress monitoring results is primarily governed by the evolution of microcracks and localized deformation within the coal–rock mass. Acoustic emission signals originate from crack initiation, propagation, frictional sliding, and local crushing, which represent microscopic damage processes. These processes lead to transient local stress redistribution around the cracks, resulting in short-term stress adjustments transmitted to the monitoring device.

As a result, increased acoustic emission activity is accompanied by enhanced high-frequency fluctuations in the monitored stress signals. However, these fluctuations mainly reflect localized and instantaneous stress adjustments associated with crack evolution, rather than changes in the global stress state. The macroscopic stress evolution captured by the monitoring device is therefore dominated by the externally applied load, while acoustic emission provides complementary information on microscopic damage development. This coupling explains the observed correspondence between intensified AE activity and stress signal fluctuations, while maintaining consistency in overall stress trends and peak responses.

### 4.2. Results of Biaxial Unequal Stress Tests

**Figure 10:**
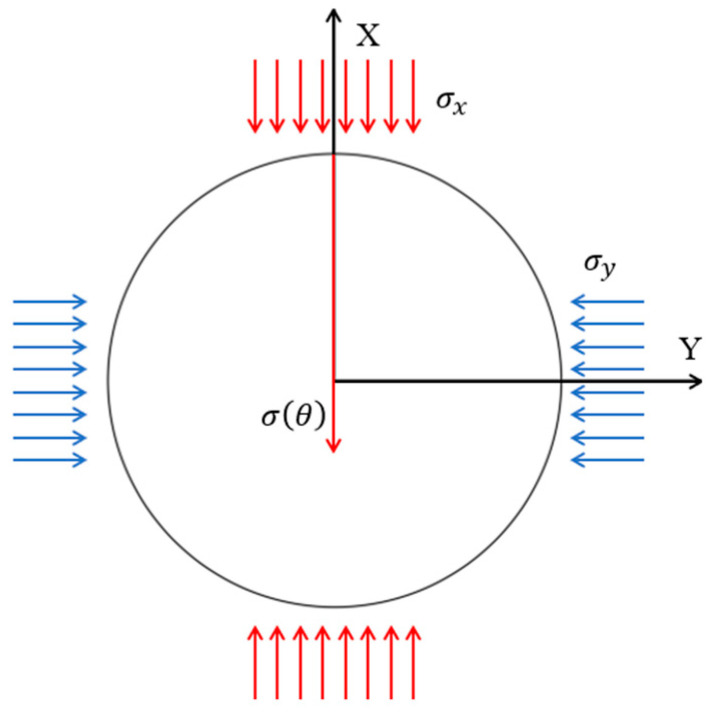
Schematic diagram of stress acting on a point within the borehole.

In the experiments, $\sigma_{x}\neq \sigma_{y}$. A Cartesian coordinate system is established with the center of the specimen as its origin. The positive *Y*-axis is defined as the 0° reference direction, and the angle (θ) is measured counterclockwise. The external loads consist of stress ($\sigma_{x}$) acting along the *X*-axis and stress ($\sigma_{y}$) acting along the *Y*-axis, with unequal magnitudes. The coordinate system is aligned with the principal stress directions, as illustrated in Figure 10.

Therefore, the shear stress component $\tau_{xy}=0$. Under two-dimensional plane stress conditions, the normal stress acting on an arbitrary direction can be expressed using the stress transformation equation as:

(11) $$\sigma_{n}(\phi )=\sigma_{x}\cos^{2}\phi +\sigma_{y}\sin^{2}\phi +2\tau_{xy}\sin\phi \cos\phi$$

where (Φ) denotes the angle between the considered direction and the positive *X*-axis. Since $\tau_{xy}=0$ in this case, the stress transformation equation can be simplified as:

(12) $$\sigma_{n}\left(\phi \right)=\sigma_{x}\cos^{2}\phi +\sigma_{y}\sin^{2}\phi$$

In this study, the positive *Y*-axis is defined as the 0° reference direction. Therefore, the angular conversion is ∅=90°−θ. Substituting this relation into the above equation and rearranging yields the stress expression in the direction of angle (*θ*):

(13) $$\sigma (\theta )=\sigma_{x}\sin^{2}\theta +\sigma_{y}\cos^{2}\theta$$

For *θ* = 0°, 120° and 240°, respectively:

(14) $$\sigma (0{}^{\circ})=\sigma_{y}$$

(15) $$\sigma (120{}^{\circ})=\frac{3}{4}\sigma_{x}+\frac{1}{4}\sigma_{y}$$

(16) $$\sigma (240{}^{\circ})=\frac{3}{4}\sigma_{x}+\frac{1}{4}\sigma_{y}$$

During the experiment, for specimen No. 5, $\sigma_{x}>\sigma_{y}.$ To achieve a biaxial unequal stress condition, different loading rates were applied along the X- and Y-axes during the initial loading stage, and loading continued until complete failure of the specimen.

**Figure 11:**
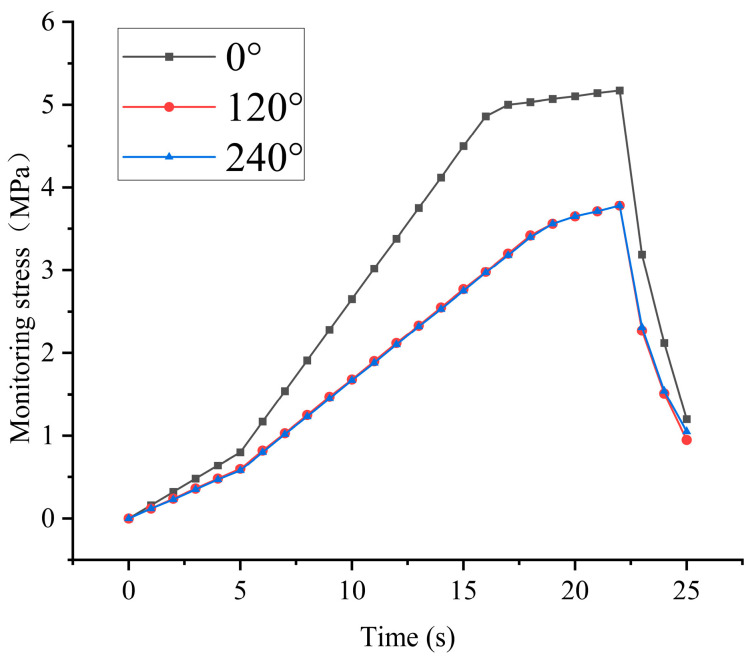
Stress monitoring results of specimen No. 5.

As shown in Figure 11. The experimental monitoring results further validate the theoretical analysis presented above. During biaxial unequal loading, the stress responses in different directions exhibit pronounced differences. The monitored stress in the 0° direction is consistently higher than that in the 120° and 240° directions, with the disparity being most significant during the late loading stage and near the peak stress. In contrast, the stress evolution trends in the 120° and 240° directions are highly consistent and show similar magnitudes, indicating comparable loading conditions along these two directions. These results demonstrate that the anisotropic stress distribution induced by non-uniform loading can be clearly identified by the in situ stress monitoring device, and that the relative magnitudes and evolution patterns of stress responses in different directions are consistent with the theoretical derivations. Therefore, the device can effectively capture the directional differences and concentration characteristics of surrounding rock stress under biaxial unequal stress conditions, confirming its reliability and applicability for in situ stress monitoring under complex stress states.

### 4.3. Integrated Discussion of Stress Monitoring Performance

By synthesizing the results obtained under different initial damage conditions and biaxial unequal stress loading, the overall performance of the developed monitoring device can be clearly assessed. The experiments consistently indicate that the device is capable of capturing the macroscopic evolution of in situ stress under both uniform and non-uniform stress fields.

Although local damage and stress anisotropy introduce increased signal fluctuations, these effects mainly reflect localized stress adjustments associated with crack activity and do not compromise the global stress evolution or peak stress identification. This demonstrates that the device can effectively distinguish between local disturbance-induced fluctuations and the overall stress response.

The integrated results confirm that the proposed monitoring approach maintains stable performance under complex stress conditions typical of deep mining, supporting its applicability for continuous stress monitoring and engineering risk assessment.

## 5. Conclusions

(1) This study developed an in situ stress monitoring device for deep mining through structural optimization and targeted material selection, aiming to address the challenges of high stress levels, strong mining disturbances, and surrounding rock degradation. Laboratory validation results demonstrate that the monitored stress remains highly consistent with the applied stress throughout the loading–unloading process, with peak errors controlled within 5% and fitting correlation coefficients exceeding R^2^ = 0.95, confirming the high accuracy and stability of the device.

(2) Experimental investigations under different initial damage conditions indicate that, although internal crack activity and signal fluctuations intensify with increasing damage, the monitored stress reliably tracks the applied stress in terms of overall evolution trend, peak stress, and unloading response. This demonstrates that the proposed device maintains stable monitoring performance under conditions involving structural degradation and complex internal disturbances.

(3) Biaxial unequal stress loading tests verify that the device can clearly identify stress directional differences, with stress in the 0° direction consistently exceeding that in the 120° and 240° directions. This confirms its capability to characterize stress directionality and concentration features under non-uniform stress fields.

Overall, this study demonstrates that the evolution of in situ stress in deep mining can be continuously and reliably captured prior to rock mass failure under strong disturbance conditions. The proposed device provides a practical pathway from discrete or static stress assessment toward dynamic and process-oriented stress perception. It offers a feasible and engineering-promising solution for quantitative monitoring of mining-induced stress evolution, with potential applications in surrounding rock stability evaluation and hazard prevention in deep underground mines.

## 6. Discussion

Several methods have been developed for in situ stress measurement in underground engineering, including overcoring techniques, hydraulic fracturing, hollow inclusion (HI) cells, and borehole stressmeters. These methods have been widely used to determine the initial in situ stress state and stress orientation in rock masses. However, most of them rely on discrete or staged measurements and are therefore less suitable for continuous stress monitoring during active mining, particularly under high-stress and strongly disturbed conditions.

In contrast, the monitoring device developed in this study is designed for borehole pre-installation and continuous recording of stress evolution during mining-induced loading and unloading processes. Although it is not intended to capture ultimate failure stresses, the device enables real-time acquisition of stress evolution, concentration, and unloading characteristics prior to rock mass failure. Moreover, it exhibits good adaptability under strong disturbance and structural degradation conditions and can be integrated with microseismic or acoustic emission monitoring systems to provide complementary information on stress evolution and damage development. These characteristics demonstrate the engineering advantages of the proposed device for in situ stress monitoring in deep mining environments.

## Figures and Tables

**Table 1:** Parameters of the foil-type strain gauge.

Model	Resistance (Ω)	Gauge Factor (GF)	Maximum Micro Strain (×10^−6^)
BX120-20AA	120.0 ± 0.3	2.08 ± 0.01	20,000
Grid material	Operating temperature range (°C)	base length × base width (mm)	grid length × grid width (mm)
Copper	−20~+80	24 × 5	20 × 3

**Table 2:** Main technical parameters.

σ_1_/MPa	σ_2_/MPa	σ_3_/MPa	Specimen Size/(mm × mm × mm)	Stress Loading Control Mode	Stress Monitoring Resolution (kPa)
8	8	8	300 × 300 × 300	Load control	0.5

## Data Availability

The raw data supporting the conclusions of this article will be made available by the authors on request.

## Abbreviations

The following abbreviations are used in this manuscript:

AE	Acoustic emission
CSIRO	Commonwealth Scientific and Industrial Research Organisation
FEA	Finite element analysis
HI	Hollow inclusion
